# The Impact of Low-Level Iron Supplements on the Faecal Microbiota of Irritable Bowel Syndrome and Healthy Donors Using In Vitro Batch Cultures

**DOI:** 10.3390/nu12123819

**Published:** 2020-12-14

**Authors:** Carlos Poveda, Dora I. A. Pereira, Marie C. Lewis, Gemma E. Walton

**Affiliations:** 1 Department of Food and Nutritional Sciences, University of Reading, P.O. Box 226, Whiteknights, Reading RG6 6AP, UK; c.g.povedaturrado@reading.ac.uk (C.P.); marie.lewis@reading.ac.uk (M.C.L.); 2 Department of Pathology, Tennis Court Road, University of Cambridge, Cambridge CB2 1QP, UK; diap2@cam.ac.uk

**Keywords:** gut microbiota, iron, irritable bowel syndrome, nanoparticulate iron, pea ferritin

## Abstract

Ferrous iron supplementation has been reported to adversely alter the gut microbiota in infants. To date, the impact of iron on the adult microbiota is limited, particularly at low supplementary concentrations. The aim of this research was to explore the impact of low-level iron supplementation on the gut microbiota of healthy and Irritable Bowel Syndrome (IBS) volunteers. Anaerobic, pH-controlled in vitro batch cultures were inoculated with faeces from healthy or IBS donors along with iron (ferrous sulphate, nanoparticulate iron and pea ferritin (50 μmol^−1^ iron)). The microbiota were explored by fluorescence in situ hybridisation coupled with flow cytometry. Furthermore, metabolite production was assessed by gas chromatography. IBS volunteers had different starting microbial profiles to healthy controls. The sources of iron did not negatively impact the microbial population, with results of pea ferritin supplementation being similar to nanoparticulate iron, whilst ferrous sulphate led to enhanced *Bacteroides* spp. The metabolite data suggested no shift to potentially negative proteolysis. The results indicate that low doses of iron from the three sources were not detrimental to the gut microbiota. This is the first time that pea ferritin fermentation has been tested and indicates that low dose supplementation of iron is unlikely to be detrimental to the gut microbiota.

## 1. Introduction

In 2015, it was reported that about a third of the World’s population suffered from anaemia, and in half of these cases, iron deficiency was the cause; there is no indication that this prevalence is decreasing [1]. Iron deficiency anaemia (IDA) is more prevalent in low-income countries, due to poor nutrient availability, reliance on plant-based foods and the impact of infection on iron absorption [2]. However, IDA is the main nutrient deficiency disorder affecting high-income countries [3]. The body absorbs iron from dietary sources; however, when dietary iron is insufficient, IDA can result, with the common symptoms being tiredness, fatigue, lethargy and breathlessness [4].

To help prevent development of IDA, low dose iron supplementation is commonplace. Iron supplements frequently take the form of ferrous (Fe^2+^) salts (e.g., ferrous sulphate, fumarate and gluconate) and ferric compounds (e.g., ferric ammonium citrate) [5]. However, solubility and bioavailability of these compounds differ; the ferrous form is more soluble and more readily absorbed, as well as cheaper to manufacture, and therefore, is the most commonly used [6,7]. Unfortunately, oral iron supplementation, particularly at high doses, is associated with negative side-effects, such as nausea, vomiting, constipation, diarrhoea, black stools and bloating, which often leads to non-adherence with a treatment plan [8,9]. In addition to the observable side effects, iron supplementation has been shown to affect the community of microbes living in the gastrointestinal tract of humans [2,10].

After consumption of macro and micronutrients, those that are not absorbed in the small intestine by the human host are available to gut microbes in the colon [11]. When high dose ferrous salt supplements are taken, the body is commonly unable to absorb all the iron and there is an increased amount that passes through to the colon [12]. Iron is essential for the growth of many microbes and when in limited supply some microorganisms do not grow optimally [13]. *Lactobacillus*, a genus associated with positive health effects, does not require iron to grow, and therefore, has a selective advantage when iron availability is low [10,14]. Conversely, when iron availability is high (e.g., following excess supplementation), it has been shown that bacterial growth is increased, including some potentially negative genera, such as *Escherichia/Shigella* and *Clostridium* [10,15]. In addition, high dose iron supplementation studies have resulted in increased gut inflammation, as demonstrated by an increase in calprotectin (protein marker of inflammation) [10,16]. The impact of iron on the gut microbiota has been reviewed in detail by Yilmaz and Li, 2018 [17]. This review indicates the potential of ferrous sulphate to promote the growth of potentially pathogenic micro-organisms. However, caution should be observed when considering findings, as many of the studies performed to date have been undertaken in infants [10,16,18,19,20], and only limited studies have investigated the effects of iron on the microbiota in adults. Adults are major supplement consumers; therefore, it is important to determine what iron does to their microbiota and whether there are less disruptive alternative supplementary approaches.

There is a symbiotic relationship between gut microbes and the human host and furthermore a bi-directional relationship between the gut microbiota and luminal iron absorption [21]. Some carbohydrates and proteins that cannot be digested and absorbed by the host in the upper gastrointestinal tract can be fermented by microbes populating the colon to produce metabolites such as short chain fatty acids (SCFA) [22]. SFCAs can be absorbed by the host, and often have positive systemic effects around the body. Protein fermentation additionally results in the production of branched-chain fatty acids (BCFA), such as iso-valerate, along with some unfavourable amino acid metabolites, such as p-cresol and indole [23]. Therefore, changes in the microbial fermentation profile can alter the bacterial metabolites produced and could impact the host [22,24]. Research of Dostal et al. (2013) showed that reducing the ferrous sulphate concentration leads to decreased levels of acetate and BCFA in vitro, which were restored in the presence of ferrous sulphate [25]. As such, this could be indicative of increased protein fermentation upon utilisation of this iron source.

Whilst the impact of iron on the microbiota has focused on ferrous sulphate at high doses, lower iron doses are often used as supplementation. Within the gut, 10–20 μM Fe represents the typical luminal concentration of iron from the diet [26,27], whilst supplementation might increase this to 50–200 μM. As such, it is important to consider disruption caused by lower, more commonly consumed supplementary doses [28].

Ferritin (an iron storage molecule) present in the diet, has been shown to be well-absorbed by the enterocytes, and therefore, may be less available to pathogenic bacteria in the distal colon [2,29]. Peas are a rich source of ferritin; however, the high phytic acid content within peas can lead to a low level of iron bioavailability [30]. Purified pea-ferritin iron could indeed provide a suitable alternative, and highly bioavailable, iron source [30,31]. The molecular structure of ferritin has provided inspiration for innovative nanoparticulate forms of iron [32,33]. These iron nanoparticles (Nano Fe(III)) mimic a ferrihydrite-like core, similar to that of ferritin but without the protein shell. The Nano Fe(III) molecule is modified by incorporating tartatic and adipic ligands into the construct (named IHAT). IHAT has been observed to be highly bioavailable in humans (~80% of ferrous sulphate), with low toxicity (in cellular and murine models), and has promoted positive bacterial changes in anaemic murine mice [32,33,34].

When investigating changes in the microbiota because of iron, it is also worth considering the health status of the test subjects. Healthy individuals have been shown over time to maintain a relatively consistent community of gut bacteria and when subjected to environmental changes (e.g., changes in nutrients or condition), they will tend towards that steady state. However, after continued exposure, the bacterial equilibrium will move away from that previously consistent community, which raises the possibility that the microbiota from those with other conditions may not respond in the same way [35]. Irritable bowel syndrome (IBS) is a relatively common, debilitating condition defined by abdominal pain, bloating and excess wind, with diarrhoea or constipation, or a combination of the two. Perturbations in the composition and/or activity of the gut microbiota are associated with IBS [36], whilst the microbial communities appear to be distinct in the two IBS sub-types: diarrhoea-predominant IBS (IBS-D) and constipation-predominant IBS (IBS-C). Therefore, there is the possibility that modulation of the microbiota by iron would be more apparent whilst using the microbiota from those with IBS.

The majority of consumed iron is not absorbed; as such, it remains available and can have an impact on the gut microbial community [21,33,34]. In vitro batch cultures held under anaerobic, pH-controlled conditions provide a useful approach to studying gut microbial communities and to determine how they are influenced by the presence of different substrates and supplements. Macfarlane et al. showed how a complex media could support the growth of gut micro-organisms to provide a microbial community similar to that within the gut of sudden death victims [37]. As such, these models provide a valuable source of experimentation, and have previously been used to identify how a microbial community is likely to respond before carrying out studies in more complex gut models or even human trials [38,39]. As such, the approach outlined in this manuscript allows exploration of how different iron sources impact on the gut microbial community.

The objective of this study was to observe the effects of three different forms of iron supplementation at low doses, namely, ferrous sulphate, pea ferritin and IHAT, on the microbiota and subsequent metabolites of healthy and irritable bowel syndrome donors using in vitro, pH controlled, anaerobic, faecal batch culture models.

## 2. Materials and Methods

### 2.1. Participants

Fifteen participants in total, aged between 25 and 52 years old, were recruited from the local Reading area (UK) to donate fresh faecal samples to use within fermentation models. There were five healthy donors (two male, three female, age range 23–38 years), five constipation-predominant IBS (IBS-C) donors (one male, four female, age range 22–55 years), and five diarrhoea-predominant IBS (IBS-D) donors (one male, four female, age range 23–50 years). Donors were excluded if they had taken antibiotics within 3 months of sample donation. Additional exclusion criteria were regular consumption of supplements containing iron, prebiotics or probiotics; consumers of medication active on the gastrointestinal tract (e.g., proton pump inhibitors) and those with gastrointestinal illness (other than IBS). IBS volunteers were diagnosed to be suffering from IBS by their GP.

### 2.2. Ethics

This study was conducted according to the guidelines laid down in the Declaration of Helsinki following Good Clinical Practice. It was approved for conduct by the University of Reading’s Research Ethical Committee (ethics reference UREC 1520). All volunteers provided informed written consent before providing stool samples.

### 2.3. Materials

Unless otherwise stated, all chemicals and reagents were obtained from Sigma-Aldrich Co Ltd. (Poole, UK). Ferrous sulphate and the Ammonia assay kit (53659 FluoroSELECT™ Ammonia Kit) were also obtained from Sigma-Aldrich Co Ltd. Pea ferritin was purified from marrowfat peas as previously described [30]. All nucleotide probes used for fluorescence in situ hybridisation (FISH) were commercially synthesised and labelled with the fluorescent dye Alexa Fluoro 488 or Alexa Fluoro 647 at the 5′ end (Eurofins, Wolverhampton, UK). Sterilisation of media and instruments was carried out by autoclaving at 121 °C for 15 min.

### 2.4. Faecal Sample Preparation

Faecal samples were collected and used in the experiment within 2 h of production. Anaerobic conditions were maintained in the collection containers through the use of anaerobic sachets (Thermo Scientific™, Basingstoke, UK, Oxoid AnaeroGen 2.5 L). Prior to use whole faecal samples were diluted 1:10 (*w*/*v*) with anaerobic phosphate buffered saline (0.1 M PBS, pH 7.4) and homogenised in a stomacher for 2 min (460 paddle beats/min). Prior to inoculation, a sample was taken to assess the baseline faecal microbial characteristics of the volunteers.

### 2.5. Faecal Batch Culture Fermentation

Fifteen separate fermentation experiments were carried out, with a sample from a different donor used for each experiment. Batch culture fermentation vessels were autoclaved and aseptically filled with 135 mL of low iron gut model medium (peptone water (10 g/L), casein (3 g/L), pectin (2 g/L), xylan (2 g/L), arabinogalactan (2 g/L), starch (5 g/L), guar gum (1 g/L), inulin (1 g/L), KH_2_PO_4_ (0.5 g/L), K_2_HPO_4_·3H_2_O (0.5 g/L), NaHCO_3_ (1.5 g/L), KCl (4.5 g/L), NaCl (4.5 g/L), MgSO_4_·7H_2_O (1.25 g/L), CaCl_2_·2H_2_O (0.15 g/L), Cysteine-HCl (0.8 g/L), ox-bile (0.4 g/L), vitamin K (10 μL/l), tween 80 (1 mL/L) and resazurin (4 mg/L of a 0.024% (*w*/*v*) resazurin solution). The mineral solution consisted of (Na_3_Citrate·2H_2_O (pH 6.5) (21 g/L), MnSO_4_·2H_2_O (5 g/L), CoSO_4_·6H_2_O (1 g/L), ZnSO_4_·7H_2_O (1 g/L), CuSO_4_·5H_2_O (1 g/L) AlK(SO_4_)_2_ (0.1 g/L), H_3_BO_4_ (0.1 g/L), Na2MoO_4_·2H2O (1 g/L), NiCl_2_·6H_2_O (0.25 g/L), Na_2_SeO_3_ (2 g/L), V(III)Cl (0.1 g/) and Na_2_WO_4_·2H_2_O (0.1 g/L)). The vitamin solution consisted of (biotin (2 mg/L), folic acid (2 mg/L), pyridoxine HCl (10 mg/L), thiamine HCl (5 mg/L), riboflavin (5 mg/L), nicotinic acid (5 mg/L), DL-calcium pantothenate (10 mg/L), vitamin B12 (0.5 mg/L), p-aminobenzoic acid (5 mg/L), lipoic acid (5 mg/L) and menadione (1 mg/L)), which was prepared and filtered (1% *v*/*v*), and each solution was added to the media in the autoclaved vessel. The media was a reduced iron version of the gut model media validated by Macfarlane et al. 1998 [37]. Anaerobic conditions were created by gassing vessels overnight with N_2_ (15 mL/min).

The vessels were maintained at 37 °C via a circulating water bath and the pH within the vessels was maintained between 6.3 and 6.5 using a pH controller connected to 0.5 N solutions of HCl and NaOH (Electrolab, Tewkesbury, UK). Immediately prior to faecal sample inoculation, test substrates were added to the vessels; these consisted of the equivalent of 50 µM iron i.e., 2.085 mg iron sulphate (ferrous II sulphate, Sigma, UK), 1.5 mL of 2.2 mg/mL pea ferritin (containing 5 mM Fe) and 1.745 mg IHAT (Nemysis, UK) or prebiotic β-galactooligosaccharide (B-GOS) (1.5 g) (Clasado, Reading, UK) and a control vessel with no additional substrate (negative). The vessels were inoculated with 15 mL of faecal slurry (1:10, *w*/*w*), and anaerobic conditions at 37 °C and pH 6.3−6.5 were maintained for 24 h to try to recreate some of the conditions between the transverse and distal regions of the large intestine. Samples were collected at three time points (0, 8 and 24 h).

### 2.6. Preparation of the Samples for Bacterial Metabolite Analysis, Ammonia Analysis and Flow Cytometry-Fluorescent In Situ Hybridisation (FISH)

Samples were taken at 0, 8 and 24 h post inoculation. A total of 1 mL was centrifuged (in a micro centrifuge tube) at 11,337× *g* for 5 min the supernatant was stored at −20 °C for metabolite analysis.

For FISH analysis 0.75 mL was placed in a micro centrifuge tube and centrifuged for 5 min at 11,337× *g*. The supernatant was removed, 375 µL PBS was added and mixed and 1125 µL 4% paraformaldehyde solution was added. The sample was stored at 4 °C for 4 h, then washed twice with PBS. The pellet was resuspended in 150 µL PBS, to which 150 µL ethanol was added, which was then mixed and stored at −20 °C, for future flow-FISH analysis. A further 1 mL sample was collected and stored at −20 °C, for ammonia analysis.

### 2.7. Flow-FISH Analysis

The samples were prepared for flow-FISH analysis as indicated by Rigottier-Gois et al. (2003) and Rochet et al. (2004) [40,41], modified for analysis with the Accuri C6 flow cytometer. Briefly, samples were removed from storage at −20 °C, and once defrosted, they were vortexed for 10 s. A total of 75 µL of the sample was added to 500 µL PBS in an Eppendorf tube (1.5 mL), vortexed and centrifuged at 11,337× *g* for 3 min. The supernatant was removed, and 100 µL Tris-EDTA buffer containing lysozyme (1 mg/mL) was added to the tube, mixed using a pipette and incubated in the dark at room temperature for 10 min. The samples were vortexed, centrifuged at 11,337× *g* for 3 min and the supernatant removed. The pellet was resuspended in 500 µL of PBS, then vortexed and centrifuged at 11,337× *g* for 3 min and lastly, the was supernatant removed.

The pellet was resuspended in 150 µL hybridisation buffer (0.9 M NaCl, 0.2 M Tris-HCl (pH 8.0), 0.01% sodium dodecyl sulphate, 30% formamide), vortexed and centrifuged at 11,337× *g* for 3 min. The supernatant was removed and pellet resuspended in 1 mL hybridisation buffer. Four µL of the oligonucleotide probe solutions (50 ng/µL) (refer to section below) was added to 50 µL of the sample in Eppendorf tubes (1.5 mL), vortexed and incubated at 36 °C overnight, conditions previously determined by ourselves to be optimum with the probes selected [42]. Following hybridisation, 125 µL of hybridisation buffer was added to each tube, vortexed and centrifuged at 11,337× *g* for 3 min. The supernatant was removed and the pellet was resuspended in 175 µL washing buffer solution (0.064 M NaCl, 0.02 M Tris/HCl (pH 8.0), 0.5 M EDTA (pH 8.0), 0.01% sodium dodecyl sulphate), vortexed and incubated for 30 min at 35 °C in the dark, to remove any non-specific binding of the probe. The samples were then centrifuged at 11,337× *g* for 3 min, the supernatant was removed and 300 µL PBS was added, then vortexed. The samples were stored at 4 °C in the dark prior to flow cytometry. Fluorescence measurements were performed by a BD Accuri™ C6 flow cytometer, BD, Erembodegem, Brussels, measuring at 488 nm and 640 nm and analysed used the Accuri CFlow Sampler software.

### 2.8. Bacterial Quantification Using Flow-Fluorescent In Situ Hybridisation (FISH)

Differences in bacterial populations were assessed using flow-FISH analysis with oligonucleotide probes designed to target specific regions of 16S rRNA. The probes used were Bif164 for *Bifidobacterium* spp. [43], Bac303 *for Bacteroides–Prevotella* group [44], Lab158 for *Lactobacillus/Enterococcus* [45], Erec482 for *Eubacterium rectale–Clostridium coccoides* group, Chis 150 for the *Clostridium histolyticum* group [46], Rrec584 for *Roseburia–E. rectale* group, Prop853 for clostridial cluster IX [47], Ato291 for *Atopobium* cluster [48], Fprau 645 for *Faecalibacterium prausnitzii* spp. [49] and Dsv687 for most Desulfovibrionales (excluding Lawsonia) and many Desulfuromonales [50]. Three probes (Eub338, Eub338II and Eub338III) formed the Eub 338 probe mix to detect the total bacterial count by targeting the bacteria domain [51]. The probe Non-Eub was used as a negative control to control for non-specific Eub338 binding [52]. The individual probe sequences can be found in Table 1.

### 2.9. Gas Chromatography (GC) for Short Chain and Branched Chain Fatty Acid Analysis

#### 2.9.1. Standard Solution Preparation

Individual solution standards at 5 mM were prepared for acetate, iso-butyrate, butyrate, propionate, valerate, iso-valerate and lactate. The external standard solution contained acetate (30 mM), iso-butyrate (5 mM), n-butyrate (20 mM), propionate (20 mM), n-valerate (5 mM), iso-valerate (5 mM) and lactate (10 mM). The internal standard consisted 2-ethylbutyric acid (100 mM) as described by Richardson et al. (1989) [53].

Briefly, 1 mL of sample was mixed with 50 µL internal standard, 0.5 mL concentrated HCl and 2 mL diethyl ether. The sample was vortexed for 1 min at 1500 rpm and then centrifuged at 865× *g* for 20 min. The top organic (diethyl ether) layer was extracted and retained; where less than 1 mL was extracted, the steps were repeated. In total, 400 µL of the ether extract was added to 50 µL MTBSTFA (N-Methyl-N-tert-butyldimethylsilyltrifluoroacetamide) and stored at room temperature for at least 48 h prior to GC analysis.

The samples were analysed using an HP GC 5690 fitted with a flame ionisation detector and a HP-5ms column (30 × 0.25 mm, 0.25 µm film thickness (Agilent, Cheschire, UK)). Injector and detector temperatures were 275 °C with the column temperature programmed to increase from 63 °C to 190 °C by 5 °C/min and held at 190 °C for 30 min. Helium was the carrier gas (flow rate 1.9 mL/min and head pressure 139.8 kPa). One µL samples were injected. Peak areas were recorded using HPChem software.

#### 2.9.2. Ammonia Assay

The procedure was followed as detailed in the FluoroSELECT™ ammonia kit instructions. Briefly, 1 mM ammonia standard was prepared by mixing 5 μL of the ammonia kit solution with 20 mM NH_4_Cl and 95 μL H_2_O in an Eppendorf tube. The working reagent was prepared by combining 100 μL assay buffer, 4 μL Reagent A (kit) and 4 μL Reagent B (kit) for each sample. To a multiwell plate, a range of calibration standards were added at concentrations of 0.1 μM to 50 μM, with the sample added to the remaining wells. To this, 100 μL working reagent was added. The plate was mixed and incubated for 15 min at room temperature in the dark. Readings were taken at 460 nm on a tecan plate reader, (Tecan, Theale, UK). The ammonia concentration of each sample was calculated from the calibration curve.

#### 2.9.3. Statistical Methods

Comparisons between substrates and within each substrate for the different time-points were carried out using repeated-measures two-way ANOVA with the Dunnett’s multiple comparisons test. Reported *p*-values are multiplicity adjusted. Statistical comparisons were performed using GraphPad for Prism 7 for Mac. Results are presented as means with standard deviations (SD), unless otherwise stated. The significance level was set at *p* < 0.05.

Beta diversity or between-community diversity indicates similarity (or difference) in organismal composition between samples and acts as a similarity (or dissimilarity) score between groups. Here, we show beta diversity using dimension reduction ordination analysis [54]. Principal component analysis was carried out using IBM SPSS Statistics 25. When comparing the full data set, an unpaired students *t*-test was used to compare results at a given time point with those of the control.

## 3. Results

### 3.1. Bacterial Population Enumeration by Flow-FISH

**Figure 1:**
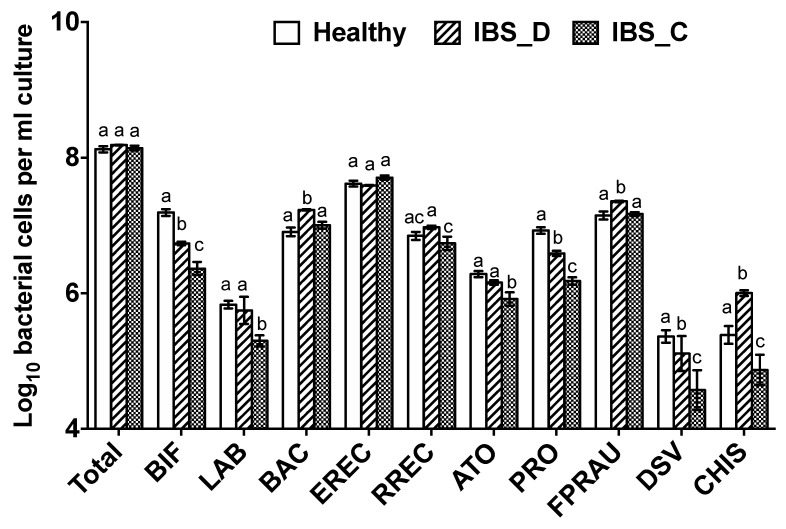
Baseline bacteria profile as detected by flow-FISH from faeces of healthy donors (*n* = 5) and IBS constipation-predominant IBS (IBS-C) (*n* = 5) and diarrhoea-predominant IBS (IBS-D) (*n* = 5) donors. Different letters indicate statistically significant differences within each bacteria group (*p* < 0.05).

Differences in bacterial populations were observed between the three different donor groups. Healthy donors had significantly more bifidobacteria (BIF) than IBS-D, who had more than IBS-C donors; the same pattern was also observed for the *Clostridium* group IX (PROP). Additionally, those with IBS-C had significantly fewer lactobacilli (LAB) than IBS-D and healthy donors. Donors with IBS-D had significantly more bacteria within the *Clostridium histolyticum* (CHIS) group within their samples (Figure 1).

**Figure 2:**
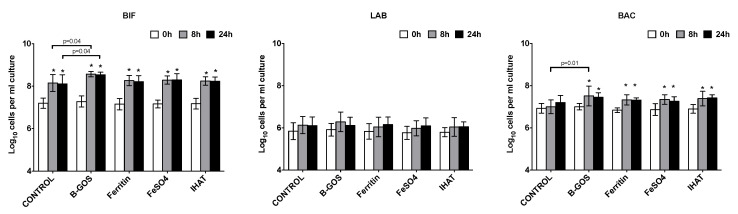

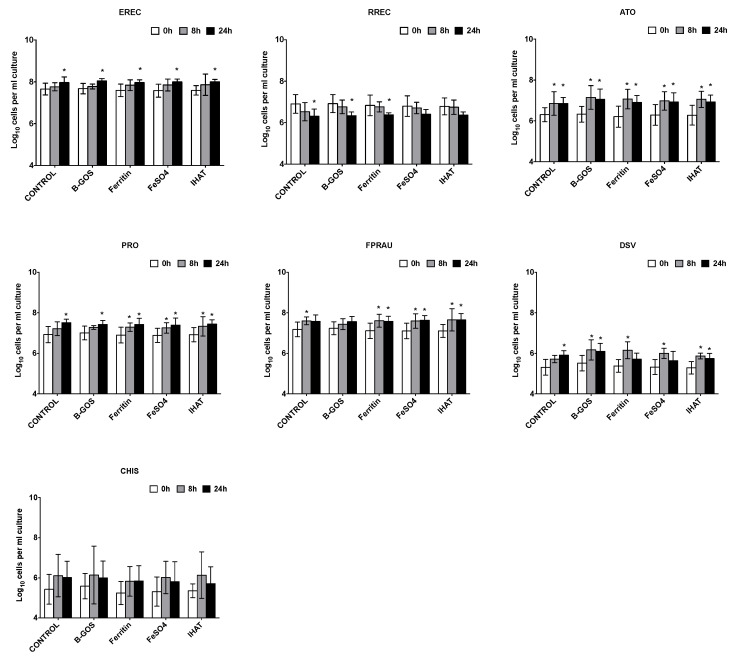
Effect of different substrates on bacterial groups detected by flow-FISH in pH-controlled batch cultures inoculated with faeces from healthy donors. Samples were collected at 0 h (baseline), 8 h and 24 h. Target bacteria: *Bifidobacterium* spp. (BIF), *Lactobacillus* spp. (LAB), most *Bacteroidaceae* and *Prevotellaceae* (BAC), *Clostridium coccoides*–*Eubacterium rectale* group (EREC), *Roseburia* subcluster (RREC), *Faecalibacterium prausnitzii* (FPRAU), *Clostridium* cluster IX (PRO), *Atopobium*–*Coriobacterium* spp. (ATO), *Desulfovibrio* (DSV) and *Clostridium histolyticum* (CHIS). Values are the mean from five independent experiments ± SD. Differences between substrates are indicated with *p*-values. *, significant differences from the baseline condition (i.e., time-point 0 h) for each substrate, *p* < 0.05.

**Figure 3:**
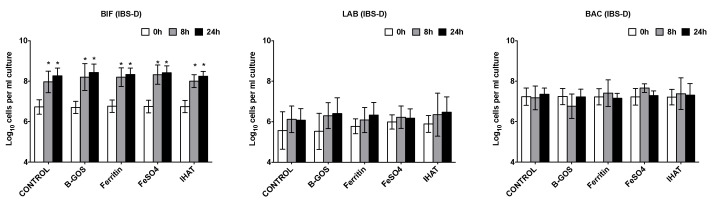

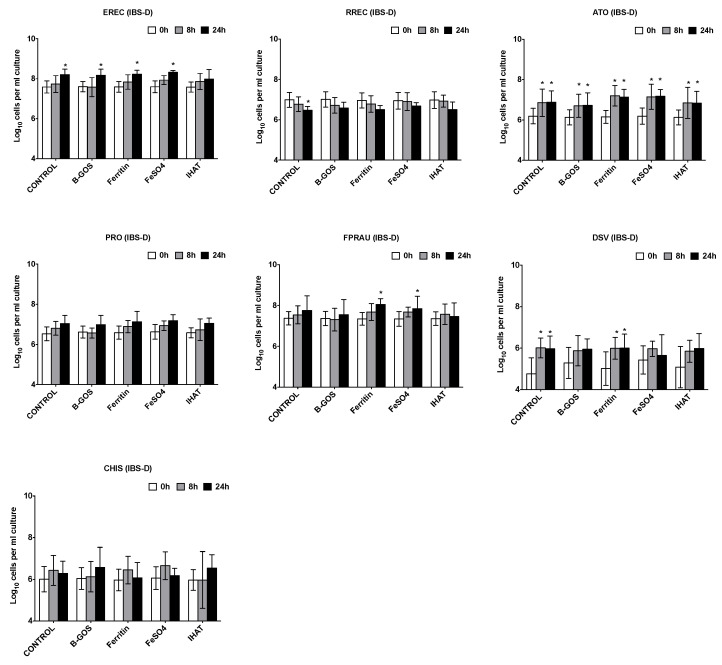
Effect of different substrates on bacterial groups detected by flow-FISH in pH-controlled batch cultures inoculated with faeces from diarrhoea-predominant IBS (IBS-D) donors. Samples were collected at 0 h (baseline), 8 h and 24 h. Target bacteria: *Bifidobacterium* spp. (BIF), *Lactobacillus* spp. (LAB), most *Bacteroidaceae* and *Prevotellaceae* (BAC), *Clostridium coccoides*–*Eubacterium rectale* group (EREC), *Roseburia* subcluster (RREC), *Faecalibacterium prausnitzii* (FPRAU), *Clostridium* cluster IX (PRO), *Atopobium*–*Coriobacterium* spp. (ATO), *Desulfovibrio* (DSV) and *Clostridium histolyticum* (CHIS). Values are the mean from five independent experiments ± SD. Differences between substrates are not statistically significant. *, significant differences from the baseline condition (i.e., time-point 0 h) for each substrate, *p* < 0.05.

**Figure 4:**
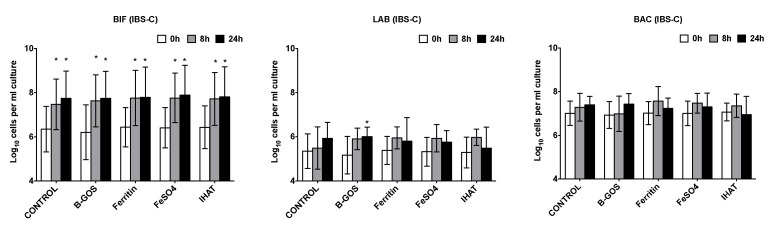

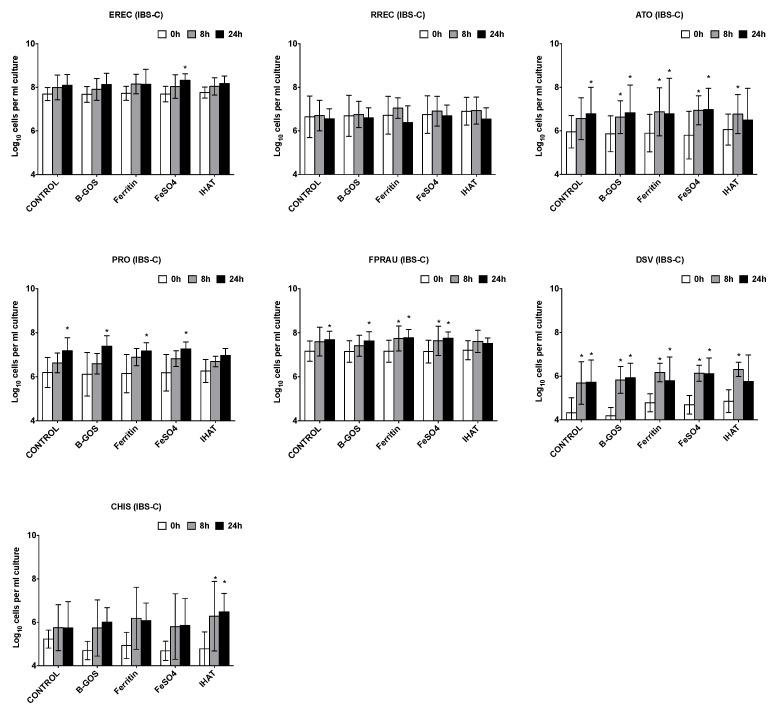
Effect of different substrates on bacterial groups detected by flow-FISH in pH-controlled batch cultures inoculated with faeces from diarrhoea-predominant IBS (IBS-C) donors. Samples were collected at 0 h (baseline), 8 h and 24 h. Target bacteria: *Bifidobacterium* spp. (BIF), *Lactobacillus* spp. (LAB), most *Bacteroidaceae* and *Prevotellaceae* (BAC), *Clostridium coccoides*–*Eubacterium rectale* group (EREC), *Roseburia* subcluster (RREC), *Faecalibacterium prausnitzii* (FPRAU), *Clostridium* cluster IX (PRO), *Atopobium*–*Coriobacterium* spp. (ATO), *Desulfovibrio* (DSV) and *Clostridium histolyticum* (CHIS). Values are the mean from five independent experiments ± SD. Differences between substrates are not statistically significant. *, significant differences from the baseline condition (i.e., time-point 0 h) for each substrate, *p* < 0.05.

Increases in total bacterial numbers were observed following all treatments and volunteer groups (*p* < 0.05) (Figure 2, Figure 3 and Figure 4). Furthermore, for all volunteer groups and treatments, significant increases in *Bifidobacterium* and *Atopobium*–*Coriobacterium* spp. (ATO) were observed following 8 and 24 h of fermentation.

Significant increases in *Clostridium coccoides*–*Eubacterium rectale* group (EREC) were observed within the healthy and IBS-D batch cultures following 24 h fermentation for all substrates, apart from IHAT, where there was just a significant increase in the case of the healthy donors (Figure 2 and Figure 3). In the presence of ferrous sulphate, the IBS-C donor samples also significantly increased in *Clostridium coccoides*–*Eubacterium rectale* (Figure 4).

The levels of *Clostridium* cluster IX increased within the vessels of the healthy and IBS-C batch cultures following 8 and 24 h of fermentation for all substrates, apart from IHAT, where there was just a significant increase in the case of the healthy donors (Figure 2 and Figure 4). There was a significant increase in the numbers of lactobacilli following the fermentation of B-GOS in the vessels inoculated with the IBS-C samples, whilst *Faecalibacterium prausnitzii* (FPRAU) levels increased after the fermentation of all substrates.

The levels of *Desulfovibrio* (DSV) were close to our limit of detection, but tended to increase during the fermentations.

When considering the healthy donors, there was also significantly more *Bacteroides-Prevotella* spp. (BAC) following fermentation of all substrates after 8 and 24 h fermentation. This was not the case for the control vessel, or for the IBS volunteer samples. Furthermore, a decrease in the *Roseburia* subcluster (RREC) was observed at 24 h across all substrates, but this only reached statistical significance in the control, B-GOS and ferritin-supplemented cultures (Figure 2).

In relation to the differences between the test substrates and the control, for healthy donors there was significantly more *Bifidobacterium* following the fermentation of B-GOS after 8 and 24 h of fermentation. There was also significantly more *Bacteroides-Prevotella* spp. (BAC) at 8 h in the batch cultures with B-GOS, compared to the control vessel. No other significant differences were detected between the test substrates (Figure 2).

In the IBS batch cultures, there were no significant differences between the test substrates for any time point of fermentation (Figure 3 and Figure 4).

**Figure 5:**
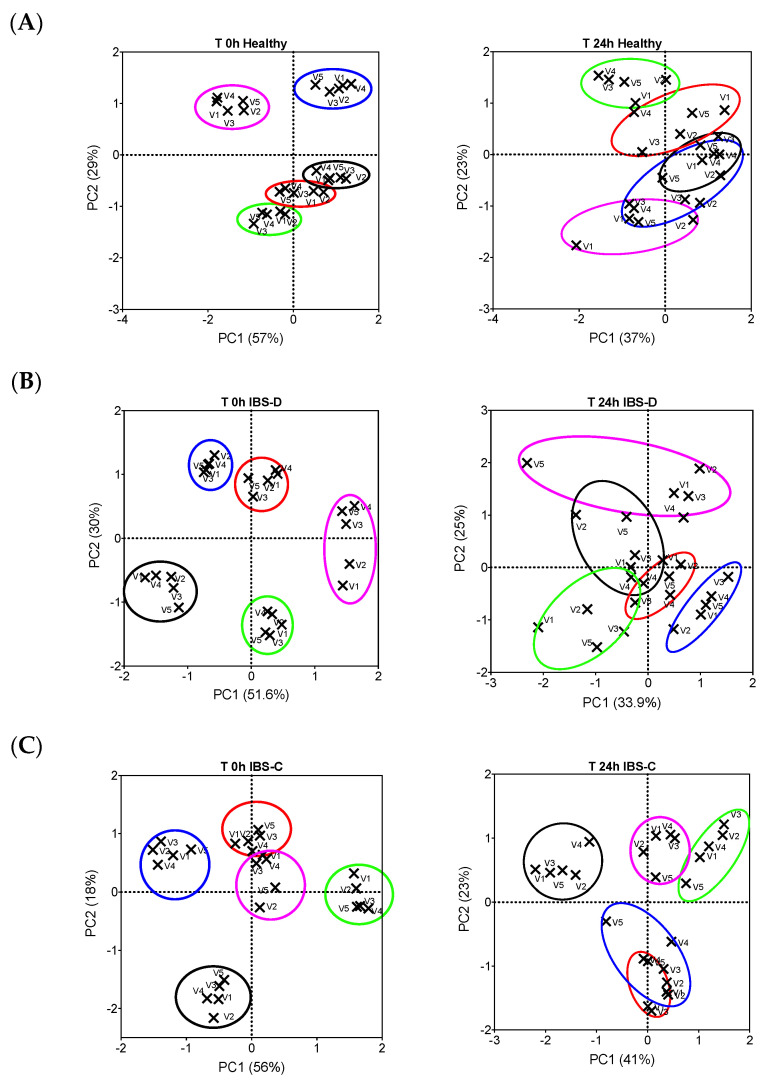
Score plot of the principal component analysis representing the variation in bacterial composition at 0 and 24 h post-inoculation with fresh faecal material collected from (**A**), healthy donors (*n* = 5); (**B**), diarrhoea-predominant IBS (IBS-D, *n* = 5) and (**C**), constipation-predominant IBS (IBS-C, *n* = 5) donors. The five independent donors are colour coded (red, blue, green, pink and black clusters) and the different experimental conditions are labelled as V1 (Control, only gut model medium), V2 (B-GOS), V3 (ferritin), V4 (FeSO_4_) and V5 (IHAT). The percentage variance values accounted for by the two first components (PC1 and PC2) are shown in parenthesis.

When all batch culture data were combined (healthy and IBS), the ferrous sulphate treatment resulted in a significantly more *Bacteroides* spp. as compared to the negative control (*p* = 0.038). A principal component score plot of between-community bacterial diversity (beta-diversity) at the start of fermentation and after 24 h showed that the clustering of samples was driven by faecal donor, rather than substrate. As such, there was no evident clustering by substrate or by the iron sources (V3–V5 in Figure 5) and non-iron sources (V1–V2 in Figure 5).

### 3.2. Bacterial Metabolites

**Figure 6:**
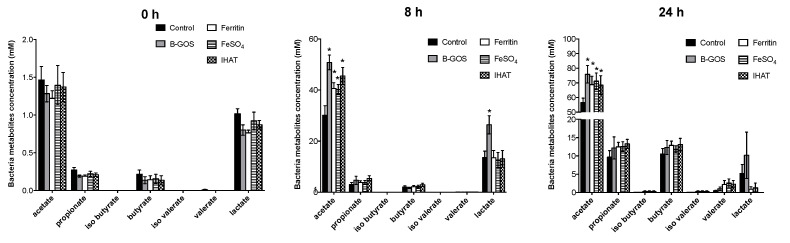
SCFA production at baseline (0 h), 8 and 24 h of fermentation in pH-controlled batch cultures inoculated with faeces from healthy adult donors. Values are mean ± SD of five independent experiments (five donors). *, significant differences from the control condition (i.e., gut model media only, with no additional test substrate), *p* < 0.05.

**Figure 7:**
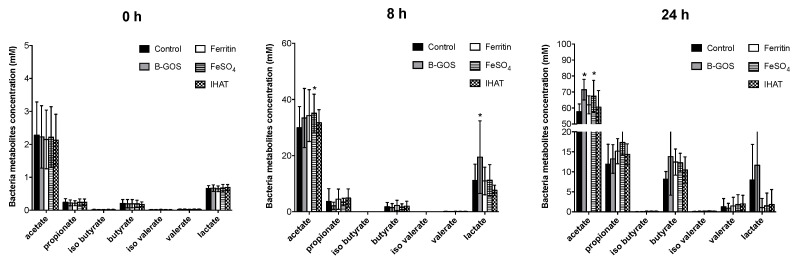
SCFA production at baseline (0 h), 8 and 24 h of fermentation in pH-controlled batch cultures inoculated with faeces from diarrhoea-predominant IBS (IBS-D) donors. Values are mean ± SD of five independent experiments (five IBS-D donors). *, significant differences from the control condition (i.e., gut model media only, with no additional test substrate), *p* < 0.05.

**Figure 8:**
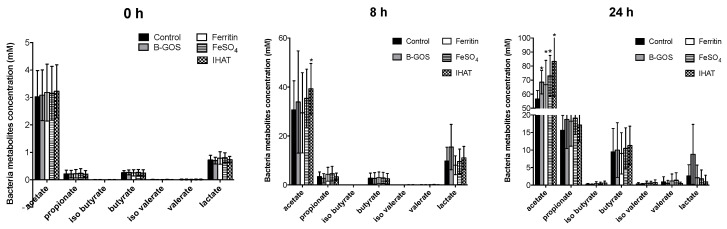
SCFA production at baseline (0 h), 8 and 24 h of fermentation in pH-controlled batch cultures inoculated with faeces from constipation-predominant IBS (IBS-C) donors. Values are mean ± SD of five independent experiments (five IBS-C donors). *, significant differences from the control condition (i.e., gut model media only, with no additional test substrate), *p* < 0.05.

Concentrations of acetate, propionate and butyrate were significantly higher following 24 h of fermentation when compared with 8 h for all conditions tested and for healthy and IBS donors (Figure 6, Figure 7 and Figure 8). Acetate was the dominant SCFA produced in all the batch cultures (Figure 6, Figure 7 and Figure 8). Branched chain fatty acids (BCFA), such as iso-butyrate and iso-valerate, indicative of proteolytic fermentation, were present at very low levels for all test conditions.

Looking at the data for volunteer groups separately, the different added test substrates did not appear to significantly impact SCFA or BCFA production by the microbiota present in the culture vessels inoculated with faecal material from healthy or IBS donors.

When viewed as a whole dataset, however, all substrates led to enhanced acetate levels (Table 2) and fermentation in the ferrous sulphate vessel led to enhanced levels of propionate (*p* = 0.027). B-GOS fermentation led to enhanced levels of lactate at 8 h (*p* = 0.007) and reduced levels of ammonia after 24 h (*p* = 0.012). Within the iron vessels, lactate levels were significantly lower than within the negative control.

**Figure 9:**
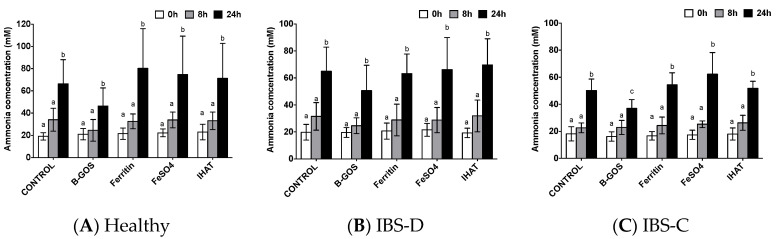
Ammonia production in pH-controlled batch cultures inoculated with faeces from (**A**), Healthy donors; (**B**), IBS-D donors and (**C**), IBS-C donors. Values are mean ± SD of five independent experiments. Different label letters (a, b) indicate significant differences between time-points. There were no significant differences between the test compounds and the control condition (i.e., gut model media only, with no additional test substrate), *p* < 0.05.

Ammonia concentration increased over time during fermentation but this was only statistically significant at the 24 h time-point (Figure 9). The only statistically significant difference between test substrates was lower ammonia production in the B-GOS condition for the IBS-C donor culture vessels after 24 h of fermentation. There were no other differences observed between the test compounds in terms of ammonia production for any of the time-points investigated.

## 4. Discussion

Low dose oral iron supplements are often taken to help avoid iron deficiency anaemia; however, previous studies have indicated that supplementation with ferrous salts can result in undesirable side-effects [8,9,10], often associated with changes to the microbiome. The current study has explored the impact of a low supplementary dose of ferrous sulphate, pea ferritin and IHAT (a nanoparticulate iron) on the faecal microbiota and subsequent metabolites using in vitro batch cultures inoculated with faecal bacteria from adults with IBS and healthy controls.

Within all experiments, it could be seen that the media used had an impact on the results. For example, for all substrates and all donor groups, increases in bifidobacteria were observed. Such a change can be explained by the complexity of the media used. The gut model media, although low in iron, contained a variety of carbohydrate and protein sources required by bacteria for growth. These conditions selected were designed to mimic conditions found within the proximal colon, and would, therefore, be likely to encourage the growth of gut bacteria [37]. This allows for better modelling of the gut community, allowing microbial changes to be assessed in the presence of a background healthy diet. However, as the conditions were plentiful large changes attributable to the iron supplementations could not be seen. The model is still a useful tool, and indicates the likely changes that might be seen whilst consuming a regular diet. An in vitro study of Kortman and coworkers (2016), using a low iron media with a different carbohydrate composition, showed that ferrous sulphate was disruptive to the microbiota [28], leading to decreases in bifidobacteria and lactobacilli; the effects observed were greater with 250 μmol^−1^ ferrous sulphate supplementation as compared to 50 μmol^−1^. In the current study, the lower dose of iron used, along with the altered media, is likely to have mitigated some of negative effects Kortman observed, supporting the idea that the lower dose of iron is less disruptive to the microbiota.

One consistent observation from in vivo studies is a reduction in commensals with low iron requirement (such as *Lactobacillus* or *Bifidobacterium* species [55]). Animal models and in vitro experiments indicate that increasing iron availability in the gastrointestinal tract may alter the gut microbiota, enabling potentially pathogenic microorganisms to have a competitive advantage [55]. In the current experiment, when the pooled data was looked at, increases in *Bacteroides*/*Prevotella* were apparent following fermentation of ferrous sulphate, but not the other iron sources. *Bacteroides* are known to be good at scavenging for iron [56]. Dostal et al. (2013) observed reductions in levels of *Bacteroides* spp. in vitro following the introduction of an iron chelator to the media [25]. Previous research of Mevisenn-Verhage et al. (1985) has shown milk fortified with iron to lead to increased levels of *Bacteroides* spp. in the faeces of infants [57]. In the current experiment, the media used was designed to be low in iron; as such, the environment would have been limiting for iron utilisers, and therefore, the addition of iron enabled their growth. Such increases were not observed in the presence of the IHAT or pea ferritin iron sources. IHAT is an engineered analogue of natural food iron (ferritin), which has recently been tested in a large Phase II clinical trial as an alternative iron supplement [58]. It is distinct from currently available oral iron supplements in that it is not soluble and does not require solubilisation in the stomach to be absorbed [33,34]. Conversely, IHAT is taken up as whole nanoparticles. This means that the unabsorbed IHAT fraction will remain nanoparticulate, suggesting no ‘free’ or labile ionic iron is released into the intestinal lumen and onto the intestinal mucosa, beyond its endocytotic absorption site in the duodenum [5,33,59]. The lack of increase in the presence of *Bacteroides/Prevotella* with IHAT and pea ferritin may suggest that lower levels of iron are available to the microbiota from these iron sources. Indeed, research by Pereira et al. (2015, 2014) in murine studies has indicated that IHAT is not available to gut bacteria when compared to ferrous sulphate, indicating that this source of iron may not be as disruptive to the microbiota as ferrous sulphate [32,34], the current gold standard for iron supplementation. The authors are not aware of previous work of the impact of pea-ferritin on the microbiota. Plant ferritin is a natural source of dietary iron, one of the main sources from a diet rich in pulses, and the results from the current study support that low-dose pea ferritin iron supplementation does not impact the gut microbiota.

The three iron supplements all led to lower levels of lactate compared to the control. This could be due to the fact that lactobacilli, a lactate producer, do not require iron for growth. As such, in the low iron condition of the control vessel (no supplementary iron) lactobacilli had a competitive advantage, whereas in the iron vessels, other members of the microbiota were able to compete, resulting in less lactate [10,14]. This does indicate a degree of available iron to the microbiota with all sources. However, the iron treatments did not have a large impact on the microbiota overall. This could in part be explained by the low dose used, which corresponds with the fact that the side-effects and gastrointestinal symptoms are observed when high dose iron supplements are administered. Typical anaemia treatment consists of 180 mg ferrous sulphate in tablet form, which is about 10 times the amount consumed during a standard meal, equating to 350 μmol^−1^ lumenal concentration [60]. The dose used in the current study, however, is relevant as a low dose supplement; if it is assumed that the iron intake per meal is 6 mg (equivalent of 18 mg/day), the jejunal lumenal iron concentration would be 35 μmol^−1^ following a meal [26], which could then be boosted by a low dose supplement leading to the 50 μmol^−1^ dose tested here. It is important to test iron at low doses to observe the effects of iron on the microbiota at the concentrations more typically consumed.

In the current study, the presence of iron (all sources) and the presence of B-GOS lead to increased levels of acetate as compared to the control. Whilst this would be expected for B-GOS fermentation due to the additional carbohydrate available, the reason for this occurrence in the iron fermentations may be different. Many organisms within the gut require iron for growth; as such, iron can enable enhanced microbial fermentation, resulting in increases in SCFA, as also observed by Dostal et al. (2013) [25]. Both proteolytic and saccharolytic fermentation can lead to increased levels of SCFA; however, in the absence of increased levels of branched chain fatty acids (BCFA), there is a likelihood that no significant rise in proteolytic fermentation occurs. This means that at the levels used, relating to a low dose supplement, the iron sources microbiota from the IBS-C, IBS-D and healthy adults did not have a negative impact on the fermentation profile.

Lee et al. (2017) looked at the impact of ferrous sulphate supplementation on those with inflammatory bowel disease (IBD) and on non-IBD anaemic controls. It was observed that the control volunteers had a more stable microbiota, whereas those with IBD experienced more changes to the microbiota when consuming iron 600 mg ferrous sulphate [61]. This is a much higher dose than what was used in the current study, but indicates that the microbiota of healthy volunteers may be more stable to iron supplementation. As such, there is merit in using volunteers in different clinical groups, who may have a different microbial profile to healthy adults, such as those with IBS. A strength of this research is the exploration of healthy adults alongside IBS-D and IBS-C donor groups. When donor pre-fermentation characteristics were looked at, different microbial patterns between the different donors were revealed; these included more bifidobacteria and lactobacilli in the healthy donors than those with IBS. This matches well with previous research, where lower levels of bifidobacteria have been observed in IBS-D and IBS-C volunteers [62,63,64,65]. Moreover, in the current study, higher levels of bifidobacteria and lactobacilli were observed in those with IBS-D, as compared to IBS-C. Interestingly, the IBS-D group had more bacteria from the *Clostridium histolyticum* group, which can include toxin producers. *Clostridium* IX were more abundant in healthy donors, which may inversely correlate to lower levels of propionate that have been observed in those with IBS [66]. However, as there were only five volunteers per group, caution must be exercised when interpreting these data.

The prebiotic B-GOS provided an additional carbohydrate source, resulting in reduced ammonia production within this fermentation. The data of Kortman et al. (2016) indicates that iron supplementation leads to increased proteolytic fermentation; furthermore, ammonia production is associated with protein fermentation, and often negative effects to the host [28]. Enhanced protein fermentation, using ammonia as a marker of this process, was not evident in the current study with the three iron sources as compared to the negative control. It is of note that fermentation of B-GOS resulted in reduced ammonia, compared to the other sources, highlighting the impact of having more fibre available.

Determination of changes in functional groups of the microbiota by Flow FISH is a useful tool for assessing the impact of treatments highlighting groups of bacteria with functional relevance. This means that the full community and changes on a species basis cannot be determined. However, within the experiment, the results provide important information about which groups of bacteria the iron sources impact on within the model. This information combined with SCFA and ammonia data can help determine whether the changes induced by the supplementation are likely to lead to positive or negative changes to the microbial community and their end products. Frequently the probe-set (of 10 bacterial groups) used by the researchers covers more than 80% of the microbiome domain bacteria as detected by EUB-338I, II and III oligonucleotide probes. In the current study, however, despite all volunteer groups (healthy volunteers, IBS-D and IBS-C) having similar total bacterial numbers, for the IBS-C group, less of the microbiome was accounted for, indicating the presence of other bacteria not covered within this assessment.

When sequencing has been applied by previous authors for healthy and IBS donors, a lower diversity has been observed in the latter group [64]. However, with the quantification of the 10 bacterial groups studied, the intra group variability observed was greater in IBS donors as compared with healthy donors. Nevertheless, following fermentation of all three groups, a similar bacterial community was observed after 24 h of fermentation. This demonstrates that the selected media composition and in vitro conditions were favourable for the growth of bacteria and boosted the analysed bacterial groups in a similar manner. Despite the richness of the media composition, which includes inulin, vessels where B-GOS was added led to a faster and significant increment in lactate, which demonstrates that the media alone was not the only factor impacting on the microbiota.

From studies on iron supplementation in IBD patients, it is clear that there is a need to find alternative iron sources or regimens that are less disruptive to the microbiota [62]. Furthermore, in murine IBD studies, it has been observed that genotype and inflammatory status influence shifts in the gut microbiota during dietary iron intake [67,68]; as such, the information taken here, whilst showing little change to the microbial community, should be further explored in vivo to determine how the health and inflammatory status of the host could further impact this.

To conclude, the microbiota of IBS donors differed in baseline populations; however, with the conditions of the experiment, these changes decreased during the fermentation. The addition of iron supplementations led to alterations to the fermentation profile, in terms of lower levels of lactate; however, the changes observed were not in combination with changes in microbial numbers. This experiment has shown that within the current model for low dietary iron supplementation and a background media, there were no adverse effects of ferrous sulphate, IHAT or pea ferritin to the faecal microbiota of healthy and IBS volunteers.

## Figures and Tables

**Table 1:** Oligonucleotide probe sequences.

Probe Name	Sequence
Non-Eub	ACTCCTAGGGAGGCAGA
Eub338 ^‡^	GCTGCCTCCCGTAGGAGT
Eub338 ^‡^	GCAGCCACCCGTAGGTGT
Eub338 ^‡^	GCTGCCACCCGTAGGTGT
Bif164	CATCCGGCATTACCACCC
Lab158	GGTATTAGCAYCTGTTTGGA
Bac303	CCAATGTGGGGGACCTT
Erec482	GCTTCTTAGTCARGTACCG
Rrec584	TCAGACTTGCCGYACCGC
Ato291	GGTCGGTCTCTCAACCC
Prop853	ATTGCGTTAACTCCGGCAC
Fprau655	CGCCTACCTCTGCACTAC
DSV687	TACGGATTTCACTCCT
Chis150	TTATGCGGTATTAATCTYCCTTT

^‡^ These probes are used together in equimolar concentrations (all at 50 ng/μL).

**Table 2:** Metabolite production at baseline (0 h), 8 and 24 h of fermentation in pH-controlled batch cultures inoculated with faeces from 15 donors (5 with IBS-D, 5 with IBS-C and 5 healthy controls, data combined).

	Time (hours)	Acetate	Propionate	Butyrate	Iso Butyrate	Iso Valerate	Valerate	Lactate	BCFA	Ammonia mM
B-GOS	0	2.20 ± 1.05	0.21 ± 0.09	0.2 ± 0.11	0.01 ± 0.01	0.01 ± 0.01	0.01 ± 0.01	0.72 ± 0.13	0.02 ± 0.03	18.9 3± 4.38
FeSO_4_	0	2.26 ± 1.08	0.23 ± 0.11	0.2 ± 0.12	0.01 ± 0.01	0.01 ± 0.01	0.01 ± 0.02	0.8 ± 0.21	0.02 ± 0.03	20.4 ± 4.27
IHAT	0	2.24 ± 1.06	0.22 ± 0.09	0.18 ± 0.11	0.01 ± 0.01	0 ± 0.01	0.01 ± 0.01	0.76 ± 0.13	0.02 ± 0.03	20.14 ± 5.29
negative	0	2.26 ± 1.01	0.24 ± 0.11	0.2 3 ± 0.10	0.01 ± 0.01	0.01 ± 0.01	0.01 ± 0.02	0.8 ± 0.20	0.03 ± 0.03	19.01 ± 4.59
pea ferritin	0	2.18 ± 1.11	0.21 ± 0.09	0.2 ± 0.12	0 ± 0.01	0.01 ± 0.01	0.01 ± 0.01	0.73 ± 0.15	0.02 ± 0.03	19.62 ± 5.01
B-GOS	8	39.37 ± 15.44	3.18 ± 2.57	1.95 ± 1.60	0.01 ± 0.01	0.01 ± 0.02	0.0 6± 0.06	20.46 ± 10.59 *	0.08 ± 0.08	23.99 ± 6.71
FeSO_4_	8	36.92 ± 8.05 *	3.96 ± 2.04	2.28 ± 1.58	0.02 ± 0.02	0.02 ± 0.03	0.1 ± 0.08	11.14 ± 5.51	0.13 ± 0.11	29.29 ± 7.36
IHAT	8	38.85 ± 9.31 *	4.55 ± 2.46	2.47 ± 1.75	0.02 ± 0.04	0.03 ± 0.06	0.09 ± 0.10	10.59 ± 5.28	0.14 ± 0.16	30.44 ± 8.69
negative	8	30.28 ± 8.71	3.39 ± 2.81	2.19 ± 1.58	0.01 ± 0.02	0.01 ± 0.02	0.09 ± 0.08	11.52 ± 5.51	0.11 ± 0.11	29.38 ± 9.47
pea ferritin	8	34.79 ± 11.42	4.24 ± 2.58	2.48 ± 1.73	0.01 ± 0.02	0.02 ± 0.02	0.09 ± 0.08	10.86 ± 5.29	0.12 ± 0.11	28.57 ± 8.67
B-GOS	24	71.96 ± 9.58 *	14.71 ± 6.73	12.03 ± 7.18	0.09 ± 0.10	0.13 ± 0.15	0.83 ± 1.02	10.2 ± 11.55	1.04 ± 1.06	44.68 ± 14.93 *
FeSO_4_	24	70.56 ± 11.81 *	16.34 ± 4.43 *	11.53 ± 3.64	0.29 ± 0.36	0.36 ± 0.41	1.95 ± 2.23	1.55 ± 2.66 *	2.59 ± 2.57	67.72 ± 24.62
IHAT	24	70.8 ± 19.00 *	14.93 ± 3.66	11.61 ± 4.15	0.34 ± 0.43	0.4 ± 0.53	1.57 ± 1.86	0.91 ± 2.38 *	2.31 ± 1.96	64.24 ± 21.95
negative	24	56.95 ± 5.56	12.39 ± 4.84	9.39 ± 4.23	0.12 ± 0.16	0.18 ± 0.22	0.89 ± 1.39	5.27 ± 6.24	1.19 ± 1.49	60.52 ± 17.46
pea ferritin	24	65.89 ± 12.21 *	15.3 ± 5.07	11.45 ± 4.24	0.27 ± 0.39	0.32 ± 0.43	1.63 ± 2.09	1.35 ± 2.49 *	2.21 ± 2.41	65.9 9± 23.85

Values are mean ± SD of 15 independent experiments. *, significant differences from the control condition (i.e., gut model media only, with no additional test substrate), *p* < 0.05.
